# The Real World Experience with Lecanemab: A US Case Study

**DOI:** 10.1002/alz70858_102096

**Published:** 2025-12-25

**Authors:** Rachel N Hart, Whoy Shang, Hannah Hust

**Affiliations:** ^1^ Norton Healthcare Neuroscience Institute, Louisville, KY, USA

## Abstract

**Background:**

Lecanemab is the first anti‐amyloid monoclonal antibody with full FDA‐approval to treat Alzheimer's disease (AD), a chronic and irreversible neurodegenerative disorder that affects cognitive function, leads to progressive loss of autonomy and the ability to perform activities of daily living. Lecanemab's efficacy profile suggest the potential to slow disease progression in early‐stage AD. This study aims to describe the perspectives of the patients with mild cognitive impairment (MCI) due to AD or mild AD, who receive treatment with lecanemab, and their care partners identified from a clinical practice site in the United States.

**Method:**

This cross‐sectional survey study will recruit patients who have been treated with lecanemab for at least six months and provide informed consent. The clinical practice is within a not‐for‐profit health care system that owns and operates nine hospitals, 10 outpatient centers, 19 immediate care centers, and has an expanded telehealth program. A self‐administered survey will be completed by patients and/or care partners during infusion visits. The survey will consist of 9 items concerning symptoms, disease‐ and treatment‐related needs, based on findings from the What Matters Most (WMM) study, and 2 questions on patient satisfaction. Response options will be on a 5‐point Likert scale and should relate to the 4 weeks prior to the time of rating. Sociodemographic and clinical data from electronic medical records will be used to describe participants. Descriptive statistics, including frequencies and percentages, will be reported.

**Result:**

All eligible patients will be given the opportunity to participate. Consenting patients and/or their caregivers will be surveyed. Data collection and analysis will be completed by June. Descriptive statistics, such as age, sex, race/ethnicity, disease stage, and mean number of infusions, will be presented for the study population.

**Conclusion:**

Findings from this survey study will evaluate patient‐reported outcomes and treatment satisfaction associated with lecanemab in real‐world settings. Understanding the impact on patients’ and care partners’ lives may inform treatment decisions.

